# Tuning photoluminescence of organic rubrene nanoparticles through a hydrothermal process

**DOI:** 10.1186/1556-276X-6-405

**Published:** 2011-06-01

**Authors:** Mi Suk Kim, Eun Hei Cho, Dong Hyuk Park, Hyunjung Jung, Joona Bang, Jinsoo Joo

**Affiliations:** 1 Department of Physics, Korea University, Anam-dong, Seongbuk-gu, Seoul 136-713, Korea; 2 Department of Chemical & Biological Engineering, Korea University, Anam-dong, Seongbuk-gu, Seoul 136-713, Korea

## Abstract

Light-emitting 5,6,11,12-tetraphenylnaphthacene (rubrene) nanoparticles (NPs) prepared by a reprecipitation method were treated hydrothermally. The diameters of hydrothermally treated rubrene NPs were changed from 100 nm to 2 μm, depending on hydrothermal temperature. Photoluminescence (PL) characteristics of rubrene NPs varied with hydrothermal temperatures. Luminescence of pristine rubrene NPs was yellow-orange, and it changed to blue as the hydrothermal temperature increased to 180°C. The light-emitting color distribution of the NPs was confirmed using confocal laser spectrum microscope. As the hydrothermal temperature increased from 110°C to 160°C, the blue light emission at 464 to approximately 516 nm from filtered-down NPs was enhanced by *H*-type aggregation. Filtered-up rubrene NPs treated at 170°C and 180°C exhibited blue luminescence due to the decrease of intermolecular excimer densities with the rapid increase in size. Variations in PL of hydrothermally treated rubrene NPs resulted from different size distributions of the NPs.

## Introduction

Optical properties of metal nanoparticles (NPs) can be controlled by their size and shape, which have been studied with respect to the surface plasmon band of the metal nanostructures [1-4]. For advanced control of optical properties, metal NPs can be oxidized, incorporate dye, or use polymers for the surface passivation [5-10]. In semiconducting silicon NPs, photoluminescence (PL) characteristics depend on the thickness of the oxidation layer [11]. Organic fluorescence particles have been intensively studied for fundamental research and applications to optoelectronics [12-15]. In organic semiconducting NPs, Nakanishi and coworkers reported that PL characteristics of perylene microcrystals were size dependent [16,17]. Variations in PL of 1-phenyl-3-((dimethylamino)styryl)-5-((dimethylamino)phenyl)-2-pyrazoline NPs resulted from various size crystals treated with various organic solvents and temperatures [18].

The π-conjugated 5,6,11,12-tetraphenylnaphthacene (rubrene) crystals showed excellent hole mobility and light-emitting characteristics [19-21]. Therefore, rubrene crystals and nanostructures have been intensively studied for optoelectronics applications [22-24]. Electrical and optical properties of rubrene nanowires have been investigated for field-effect transistors and optical waveguides [25-27]. However, the luminescence characteristics and their tuning of rubrene NPs have not been studied thoroughly. In this study, we introduce a hydrothermal process for control of the PL characteristics of organic rubrene NPs. Hydrothermal processes have been used for crystallization of amorphous materials, fabrication of new materials, and easy tuning of intrinsic properties in aqueous solution [28-30]. For example, bulk MgO was converted to Mg(OH)_2 _nanoplates with a hydrothermal method involving a heterogeneous reaction in aqueous media above 100°C [30].

We fabricated pristine rubrene NPs using a simple reprecipitation method. The color of light emission of the rubrene NPs changed from yellow-orange to blue with increasing hydrothermal temperatures. The diameters of filtered-up rubrene NPs increased from 350 to 890 nm with increasing hydrothermal temperatures, while those of filtered-down rubrene NPs were almost unchanged at approximately 120 nm. Hydrothermally treated (HT) rubrene NPs have size-dependent PL characteristics. Luminescence color and relative dominance of PL peaks at 464 nm to approximately 516 and 560 nm varied, depending on the hydrothermal temperature. As the hydrothermal temperature increased from 110°C to 160°C, the blue light emission at 464 to approximately 516 nm from filtered-down NPs was enhanced by *H*-type aggregation, which was supported by the optical absorption spectra. Filtered-up rubrene NPs treated at 170°C and 180°C exhibited blue luminescence due to the decrease of intermolecular excimer densities with the rapid increase in size.

### Experiment section

Pristine rubrene NPs were prepared by a conventional reprecipitation method [5]. Rubrene powder was purchased from Sigma-Aldrich Co. and used without further purification. The pristine rubrene NPs were treated hydrothermally for 10 h using a hydrothermal autoclave (Parr Instrument Acid Digestion Bombs, 4744 General Purpose Bomb, Parr Instrument Company, Moline, IL, USA). Hydrothermal treatment occurred at 110°C, 130°C, 140°C, 150°C, 160°C, 170°C, and 180°C with samples denoted HT-110, HT-130, HT-140, HT-150, HT-160, HT-170, and HT-180, respectively. During the hydrothermal process, external pressure was applied to the rubrene NPs. After the hydrothermal treatment, the hydrothermal chamber was slowly cooled at room temperature (RT). Pristine and HT rubrene NPs were centrifugally filtered (low-binding durapore PVDF membrane, Millipore Corporation, Billerica, MA, USA), with a membrane pore size of approximately 220 nm. After filtration at 5,000 rpm for 2 min, the NPs were deposited in the upper and lower parts of the filter device. Filtered-down NPs were obtained directly from the lower part of the filter. For the filtered-up NPs, 1 ml of distilled water was dropped onto the upper part of the device, and then the NP solution was sonicated for 5 min. The rubrene NPs were dried on a glass substrate in a vacuum oven for 2 h at RT.

Formation of rubrene NPs was investigated using a field-emission scanning electron microscope (SEM; JEOL KSM-5200, JEOL Ltd., Tokyo, Japan) and a high-resolution transmission electron microscope (HR-TEM; JEOL JEM-3010, JEOL Ltd., Tokyo, Japan). Size distributions of the rubrene NPs, which were homogeneously dispersed in distilled water, were measured by dynamic light scattering (DLS; BI-200SM, Brookhaven Instruments Co., Holtsville, NY, USA). For the optical properties of the rubrene NPs, ultraviolet and visible absorption (UV/vis; Agilent HP-8453 UV/vis absorption spectrophotometer, Agilent Technologies, Santa Clara, CA, USA) and PL spectra (Hitachi F-7000, Hitachi High-Technologies Co., Tokyo, Japan) in solution were measured at RT. The confocal laser spectrum microscope (CLSM, LSM 5 Exciter, Carl-Zeiss, Göttingen, Germany) was used to investigate the red (R), green (G), and blue (B) color distribution of luminescence.

## Results and discussion

### Unfiltered rubrene NPs

**Figure 1:**
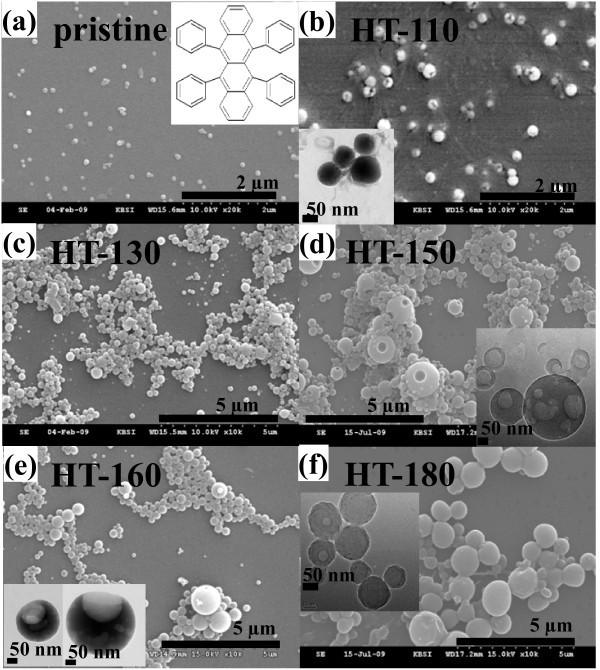
**SEM images**. (**a**) Unfiltered pristine and (**b**) HT-110, (**c**) HT-130, (**d**) HT-150, (**e**) HT-160, and (**f**) HT-180 rubrene NPs. Inset of Figure 1a: Schematic chemical structure of rubrene molecule. Insets of Figure 1b, d, e, and f: HR-TEM images of corresponding HT rubrene NPs.

Figure 1a, b, c, d, e, f and their insets show the SEM and HR-TEM images of the unfiltered pristine and HT rubrene NPs, respectively. Pristine NPs were spherical with diameters of 100 nm to approximately 200 nm (Figure 1a). The diameters of HT-110 rubrene NPs were 100 nm to approximately 250 nm (Figure 1b), and some had a nanohole of ≤20 nm on the surface. The inset of Figure 1b shows an HR-TEM image of HT-110 rubrene NPs with nanoholes. As shown in Figure 1c, HT-130 rubrene NPs have diameters of 100 nm to approximately 500 nm, some with nanoholes on the surface. We can suggest that the formation of nanoholes on the rubrene NPs might be due to the aggregation of the pristine NPs during the hydrothermal process, in which the empty spaces between the NPs could be existed and induced the nanoholes [30]. Diameters of the HT-150 NPs were 100 nm to approximately 900 nm (Figure 1d). The shapes of HT-160 and HT-180 rubrene NPs were similar to those of HT-150 NPs, and their diameters increased to 100 nm to approximately 900 and 200 nm to approximately 2 μm, respectively (Figure 1e, f). The average diameters of the unfiltered HT rubrene NPs were increased with increasing hydrothermal temperatures.

**Figure 2:**
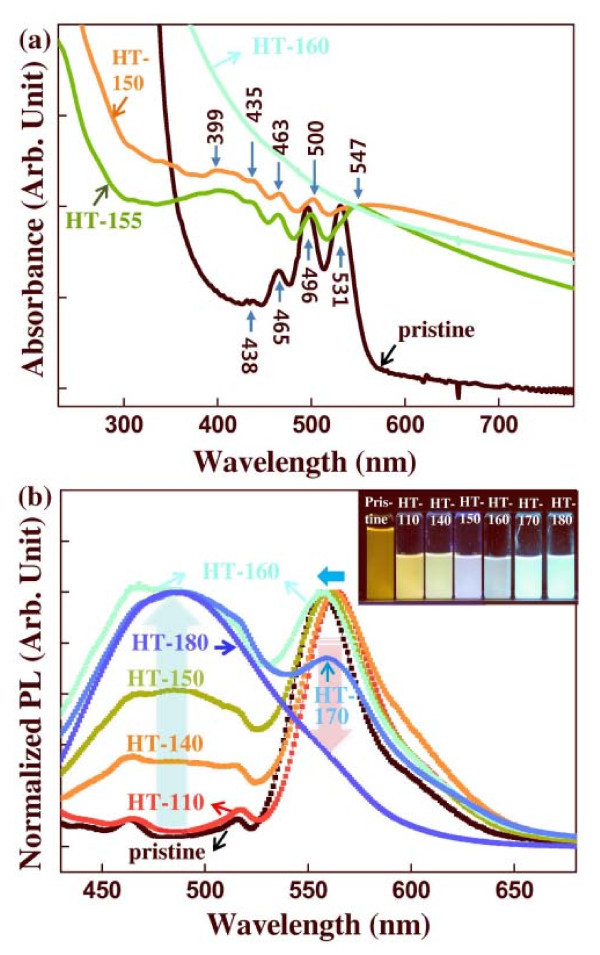
**(a) UV/vis absorption and (b) normalized PL spectra of the unfiltered pristine and HT NPs**. Inset: Photographs of light emission for the pristine and HT rubrene NPs.

Figure 2a, b shows UV/vis absorption and normalized PL spectra, repectively, of the unfiltered pristine and HT rubrene NPs. The UV/vis absorption peaks of pristine NPs were observed at the 438, 465, 496, and 531 nm, as shown in Figure 2a. In the case of the HT-150 and HT-155 NPs, the absorption peaks were observed at 435, 463, 500, and 547 nm, and new broad absorption band was appeared at approximately 399 nm (Figure 2a). The absorption peaks at 438 and 465 nm were slightly blue shifted to 435, 463 nm, respectively. The blue-shift of the absorption peaks and new absorption band at approximately 399 nm might be due to the *H*-aggregation [31], which will be discussed more detail in PL properties of the filtered rubrene NPs. For the HT-160 rubrene NPs, the UV/vis absorption characteristic peaks were disappeared.

The inset of Figure 2b is the photographs of light emission for pristine and HT NPs. Luminescence color varied from orange-yellow for pristine rubrene NPs to blue for HT-180 rubrene NPs. For pristine rubrene NPs, PL characteristic peaks were observed at 464, 516, and 556 nm. The main PL peak of bulk rubrene single crystals was observed at 570 nm, due to the *M*-axis polarized band of a short tetracene backbone in the rubrene molecules [25]. The main PL peak of the pristine rubrene NPs studied here was slightly blue shifted and observed at 556 nm, which has been also observed other NPs [32-34]. The weak PL peaks of the pristine rubrene NPs were observed at 464 and 516 nm, resulting from the PL peaks of tetracene monomers in the rubrene molecules (inset of Figure 1a) [35]. These PL peaks at 464 and 516 nm were only observed for the NP structure, not detected for bulk rubrene crystals or thin films.

The PL characteristics and their relative intensities of HT-110 rubrene NPs were similar to the pristine sample. As hydrothermal temperatures increased, the relative dominance of the PL peaks at 464 and 516 nm gradually increased and broadened for HT-140, HT-150, and HT-160 rubrene NPs, as shown in Figure 2b. The main PL peak at 556 nm for pristine rubrene NPs was blue shifted to 563, 560, and 557 nm for the HT-140, HT-150, and HT-160 samples, respectively. For the HT-170 NPs, the PL peak at 560 nm decreased, while that at 464 nm to approximately 516 nm was considerably enhanced (Figure 2b). The dominant PL peak of the HT-170 rubrene NPs was observed at 464 nm to approximately 516 nm. Eventually, for the HT-180 rubrene NPs, the PL peak at 556 nm disappeared and the broad main PL peak was observed at 487 nm, as shown in Figure 2b. We infer that PL characteristics of rubrene NPs are related to size distributions that can be controlled by hydrothermal treatment temperature. The characteristic crystalline peaks of rubrene were not observed for the pristine and HT rubrene NPs under X-ray diffraction (not shown here) patterns, indicating the amorphous phase of all rubrene NPs studied here. The results of the PL spectra of the unfiltered rubrene NPs suggest the tuning of luminescence color through the hydrothermal process.

### Filtered rubrene NPs

**Figure 3:**
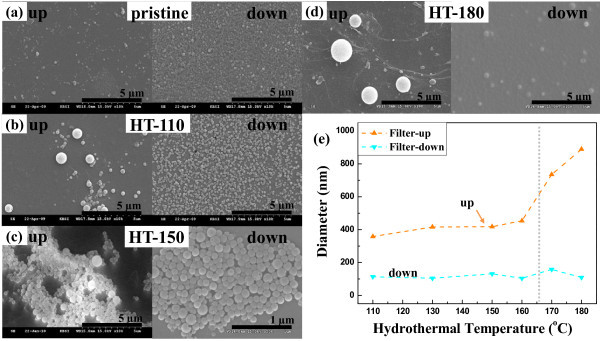
**SEM images**. The filtered-up and filtered-down (**a**) pristine, (**b**) HT-110, (**c**) HT-150, and (**d**) HT-180 rubrene NPs. **(e) **Diameters of the filtered-up and filtered-down pristine and HT rubrene NPs as a function of hydrothermal temperature.

Figure 3a, b, c, d shows SEM images of the centrifugally filtered pristine and HT rubrene NPs. Filtered-up rubrene NPs have varying diameters depending on hydrothermal temperatures. The average diameters of the filtered-up and filtered-down pristine rubrene NPs were about 170 and 120 nm, respectively. Filtered-down rubrene NPs had homogeneous size distributions. Diameters of the filtered rubrene NPs after the hydrothermal treatment were precisely measured by DLS experiments, using a syringe filter with pore size of 1 μm for the elimination of dust, as shown in Figure 3e. Filtered-down rubrene NPs had average diameters of 120 nm (± 110 nm), which were almost independent of hydrothermal temperature. Mean diameters of filtered-up rubrene NPs slightly increased from approximately 350 nm to approximately 450 nm as hydrothermal temperatures increased from 110°C to 160°C, and those of the filtered-up HT-170 and HT-180 rubrene NPs rapidly increased to 740 and 890 nm, respectively. The rapid increase in mean diameters for the filtered-up HT rubrene NPs above 160°C might correlate with the decrease of the PL peak at 560 nm shown in Figure 2b.

**Figure 4:**
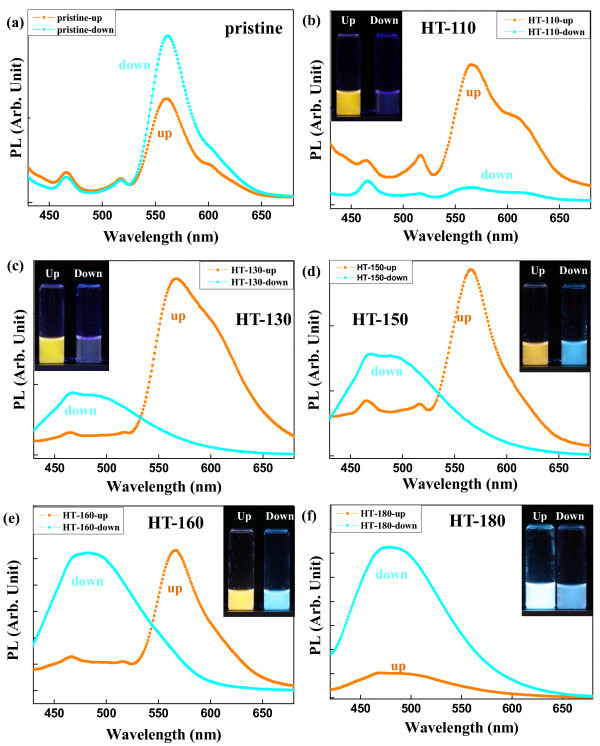
**PL spectra**. The filtered-up and filtered-down (**a**) pristine rubrene NPs and (**b**) HT-110, (**c**) HT-130, (**d**) HT-150, (**e**) HT-160, and (**f**) HT-180 rubrene NPs. Insets: Photographs of light emission for the corresponding rubrene NPs.

PL spectra of the centrifugally filtered rubrene NPs are shown in Figure 4. The insets of Figure 4 shows photographs of light emission from the filtered rubrene NPs. For the pristine NPs, the main PL peaks of both filtered-up and filtered-down NPs were at 556 nm with weak PL peaks at 464 and 516 nm, as shown in Figure 4a. For the filtered-up HT-110 rubrene NPs, the main PL peak was at 556 nm with shoulder peaks at 464, 516, and 610 nm. PL intensities of filtered-down HT-110 NPs were much weaker than those of filtered-up NPs, as shown in Figure 4b. For the HT-130, HT-150, and HT-160 rubrene NPs, contributions of the filtered-up and filtered-down NPs to the PL spectra were clearly divided into two wavelength regions, i.e., 464 nm to approximately 516 and 560 nm, as shown in Figure 4c, d, e. Filtered-up HT-130, HT-150, and HT-160 rubrene NPs had yellow luminescence, while the filtered-down samples were blue, as shown in the insets of Figure 4c, d, e. As hydrothermal temperatures increased from 110°C to 160°C, PL peaks at 464 nm to approximately 516 nm became dominant for the filtered-down rubrene NPs, as shown in Figure 4c, d, e. The enhancement of the PL peaks at 464 nm to approximately 516 nm for the filtered-down samples originated from molecular-level aggregation in the nano-size particles. Variation in optical properties of organic NPs has been reported in terms of *H*-type or *J*-type aggregation [31,36-39]. *J*-type aggregation, representing a head-to-tail molecular arrangement, induces red shift in PL by enhancement of fluorescence emission intensities [36,37]. *H*-type aggregation, representing a face-to-face packing (π-π stacking) molecular arrangement, induces blue fluorescence emission as a result of enhanced intermolecular interactions [38,39]. The degree of condensation and intermolecular interaction of rubrene molecules increased with increasing hydrothermal temperature, because external high pressure was applied to the NPs during the hydrothermal process. This process leads to generate new optical absorption band at approximately 399 nm supported by the UV/vis absorption spectra in Figure 2a, and increase the relative PL intensity at 464 nm to approximately 516 nm, which indicate the formation of *H*-aggregation [31,38,39]. Therefore, for the filtered-down rubrene NPs, the relative PL intensity at 464 nm to approximately 516 nm caused by the tetracene backbone monomer in the rubrene molecules increased with increasing hydrothermal temperature, as a result of *H*-aggregation. In the filtered-up samples, PL peaks at 560 nm decreased as diameters of the HT rubrene NPs increased. The decrease in PL intensities of organic nanostructures at longer wavelengths (≥550 nm) can be interpreted in terms of the decrease of the density of excimers [40,41]. The decrease of specific surface area with increasing particle sizes reduced the density of intermolecular excimers [40]. With increasing hydrothermal temperature for the filtered-up rubrene NPs, the diameters were increased, and the density of excimers due to the molecular packing was reduced, resulting in a decrease in the main PL peak at the 560-nm wavelength. Therefore, for the HT-180 rubrene NPs, the PL peak at 487 nm due to the filtered-up samples has been dominated, as shown in Figure 4f.

**Figure 5:**
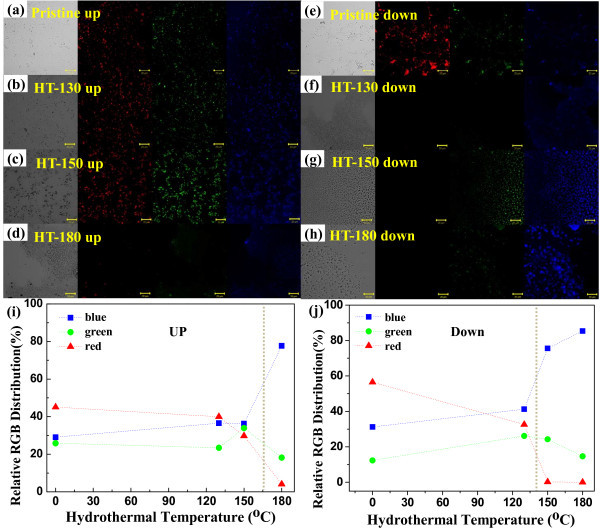
**CLSM images**. (**a**)-(**d**) CLSM images of the filtered-up pristine and HT rubrene NPs. (**e**)-(**h**) CLSM images of the filtered-down pristine and HT rubrene NPs. (**i**) Color distribution of the filtered-up pristine and HT NPs as a function of hydrothermal temperature. (**j**) Color distribution of the filtered-down pristine and HT NPs as a function of hydrothermal temperatures.

The evolution of PL characteristics of rubrene NPs through the hydrothermal process was confirmed by CLSM. Figure 5a-d and 5e-h are CLSM images for filtered-up and filtered-down rubrene NPs, respectively. For pristine NPs, the red (R), green (G), and blue (B) luminescence color distributions are 45.08%, 25.86%, and 29.06% for the filtered-up samples and 56.48%, 12.33%, and 31.19% for the filtered-down ones, respectively. Red luminescence dominated for both kinds of pristine NPs. These results are qualitatively consistent with PL characteristics shown in Figures 2b, 4a. The distribution of green luminescence for all filtered-up and filtered-down rubrene NPs were 18% to approximately 34% and 15% to approximately 26%, respectively, as shown in Figure 5i. As shown in Figure 5i, the distributions of red and blue luminescence abruptly changed for the HT-160 and HT-170 rubrene NPs, indicating the transition temperature for PL characteristics of HT rubrene NPs is 160°C to approximately 170°C. This transition temperature corresponds to the rapid variation in diameter of HT rubrene NPs, shown in Figure 3e. For filtered-up HT-180 rubrene NPs, blue luminescence increased to 78%, while that of red decreased to 18%, as shown in Figure 5i. For filtered-down rubrene NPs, blue luminescence increased from 31% in the pristine samples to 85% for the HT-180 ones, while that of red decreased from 56% in the pristine samples to 0% in the HT-180 ones. For both filtered-up and filtered-down HT-180 rubrene NPs, the dominance of blue luminescence agreed with the PL properties shown in Figure 4f.

## Conclusions

Pristine rubrene NPs prepared by reprecipitation were hydrothermally treated. The HT rubrene NPs have different size distributions depending on treatment temperature. The sizes of filtered-down rubrene NPs after the hydrothermal treatment were relatively homogeneous, with a mean diameter of approximately 120 nm. Diameters of filtered-up rubrene NPs increased from 350 to 890 nm as hydrothermal temperatures increased from 110°C to 180°C. The PL peaks of the filtered-up and filtered-down rubrene NPs, at hydrothermal temperatures from 110°C to 160°C, were observed at 560 nm (yellow-green light emission) and 464 nm to approximately 516 nm (green-blue light emission), respectively. With increasing temperature from 110°C to 160°C, the green-blue light emission became dominant for the filtered-down NPs due to the *H*-aggregation. From the UV/vis absorption spectra, the HT-150 and HT-155 rubrene NPs have new absorption band at approximately 399 nm, supporting by the formation of *H*-aggregation. Above 160°C, the filtered-up rubrene NPs exhibited blue luminescence because of the decrease of excimer density with increasing size. Color distributions for the rubrene NPs in the CLSM images qualitatively agreed with PL characteristics. Hydrothermal processing is a promising post-manipulation technique to control PL characteristics of π-conjugated organic nanostructures.

## Competing interests

The authors declare that they have no competing interests.

## Authors' contributions

MSK fabricated the pristine and HT rubrene NPs and performed the SEM, HR-TEM, and PL experiments. EHC and DHP supported the fabrication of the NPs and PL experiments. HJ and JB performed DLS experiments. JJ analyzed the results. All authors read and approved the final manuscript.
